# Intelligence

**Published:** 1808-02

**Authors:** 


### INTELLIGENCE.

We have received the following account of a new method of preparing calomel in the Laboratory of Messrs. HOWARD and Co. at Plaistow.—In their method of preparing *Calomel*, the defective operation of levigating (or in fact of grinding that solid compound in a mill, as colours are ground) with water, is wholly omitted.—They take the compound previously formed as usual, and by a sublimation, or distillation from a kind of crucible, laid horizontally in the fire, with its mouth projecting through the side of the furnace, and throw the Calomel out in vapour, which is received on the surface of water, in a veesel closely fitted to the mouth of the crucible. By this simple contrivance, they obtain the product in a state of greater whiteness, and much greater attenuation than the most assiduous levigation could effect, yet without any further change in its medicinal qualities, than may reasonably be expected to be made *for the better*, by reducing it to a state of the most minute and uniform division. It becomes, in consequence, about $\frac{2}{5}$ths lighter, or 3 lbs. occupy the same space as 5 lbs. of the levigated sort. The experience hitherto obtained of its manner of operating, justifies our expectation of its becoming a valuable addition to the Pharmacopœia. They propose to distinguish it by adding the term *Hydrosublimatus*, (which we think, though not quite classical, the most descriptive and appropriate) to the old name.——The invention is due to Mr. Joseph Jewell, late foreman of the Laboratory, and now a partner in the firm of Howard and Co. on whose account Mr. Allen, Chemist, of Plough-court, Lombard-street, will supply any medical gentlemen who may apply, with specimens; which, however, Messrs. Howard and Co. will probably distribute with a circular letter, addressed to the Faculty of the metropolis.

In the Bakerian Lecture for 1806, Mr. DAVY illustrated some important chemical agencies of electricity. In this interesting communication, some new and general philosophical principles are developed and elucidated, by a great number of minute and accurate experiments. The first principle is, " that all bodies capable of entering into chemical combination, are in opposite electrical states; or (to use the words of the author) have opposite electrical energies of these states, or energies being more exalted in proportion to the degree of affinity; and hence attraction and union would take place between them, independent of any other power than electricity. 2. That bodies which do not combine, are uniformly found, by the most delicate instruments, to exhibit the same electrical states or energy; so that, on common electrical principles, they cannot extract, but may repel each other. 3. That bodies may have their affinities increased, modified, or destroyed, by an alteration of their electrical states by artificial means. The first principle seems demonstrated by every fact advanced, nor is there one anomally. Zinc and copper by contact, are, the one positive, the other negative, according to Volta's experiments, and they combine bine when heated. The case is similar with gold and mercury, with silver and mercury, with tin and copper, and with all other metals that enter into chemical union. Oxygen and acids, Mr. Davy finds, are naturally negative; hydrogen and inflammable bodies, in general, and alkalies, positive. The Author illustrates the second principle by experiments on solid acids, metals, and alkaline earths, which do not enter into combination; and he finds that they do not exhibit any electrical energies with regard to each other, when brought in contact. The demonstration of the third principle, which is in the highest degree interesting and important, occupies by far the largest part of the paper, and has led to some very astonishing results. By having its positive electricity artificially excited, a metal is made to quit its oxygen, and the acid in which it was dissolved. When an alkali is rendered negative, it refuses to combine with acids; and acids, when made positive, refuse to combine with alkalies. The acids and alkalies in neutral compounds, are separated from each other by being placed in the Voltaic circuit: and so great is the power of electrical decomposition, that it acts upon insoluble as well as soluble materials. Glass, sulphate of barytes, sulphate of stronites, stones containing alkalies or acids, are all decomposed by electricity. Mr. Davy makes a number of important applications of his principles, and he states that there is every reason to believe that the true elements of bodies must be ascertained by means of these new powers of analysis; as their electrical energies must be limited, whereas the strength of our artificial instruments is capable of indefinite increase. This last idea he has lately himself verified to a great extent, by the decomposition of the fixed alkalies and some of the alkaline earths, a discovery of the highest importance to the progress of science.

The Royal Society has given the Copleian Medal to Mr. Home; and the president, in delivering it, took a brief, but perspicuous retrospect of the philosophical labours and discoveries in physiology by that gentleman, from the commencement of his professional career, as the successor of the celebrated John Hunter.

Mr. Carlisle, in the Croonian Lecture, which he read before the Royal Society this season, took a physiological view of the Circulation of the Blood, and of the Influence of the Nerves, so far as they operate on the muscular fibre. He then noticed the existence of an oxide of Iron discovered in the red globules of the blood, which he considered as materially influencing the muscular fibre, and the healthful state of the animal economy, and proceeded to relate the results of numerous experiments on vegetable and animal substances, in all of which he found an oxide of iron, as in peas, yolks of eggs, bile, urine, &c. The yolks of eggs he discovered to be entirely composed of a fatty oil and an oxide of iron.

The Medical and Chirurgical Society of London will shortly publish a small Selection of the most interesting Papers on Subjects relating to Medicine and Surgery, which have been read at the meetings of the Society during the last two years.

Dr.

Dr. Uwins, of Aylesbury, intends shortly to publish a small tract, entitled Modern Medicine, which will contain a familiar explanation of the most prominent discoveries and doctrines that have conduced to the recent advancement of medical philosophy; a critical disquisition on the mode in which medicine is cultivated and practised in the present period; and an inquiry how far the principles upon which the healing art is founded, may with propriety constitute a subject of unprofessional research.

Dr. Carpenter, of Exeter, is preparing for publication, an Account of the Structure and Function of the Eye, principally intended to illustrate the argument contained in the first and second chapters of Paley's Natural Theology.

A work, entitled the Medical Mentor, or Reflections on the History, Importance, Objects, and Difficulties of the Healing Art, has recently been put to press; it consists of a series of Letters from an old physician to his son, during his College and other studies, preparatory to his engagements in the active duties of his profession, and comprises a history of physic, a view of the present state of medicine and medical practitioners; an account of the qualifications necessary for the profession, and a general view of the education and preparatory studies best adapted to qualify the pupil for the discharge of its duties.

The Spring Course of Lectures at the adjoining Hospitals of St. Thomas's and Guy's, will commence the beginning of February, viz. St. Thomas's, Anatomy and the Operations of Surgery, by Mr. Cline and Mr. Cooper. Principles and Practice of Surgery, by Mr. Cooper.——Guy's. Practice of Medicine, by Dr. Babington and Dr. Curry. Chemistry, by Dr. Babington, Dr. Marcet, and Mr. Allen. Experimental Philosophy, by Mr. Allen. Theory of Medicine, and Materia Medica, by Dr. Curry and Dr. Cholmley. Midwifery, and Diseases of Women and Children, by Dr. Haighton, Physiology, or Laws of the Animal Œconomy, by Dr. Haighton. Structure and Diseases of the Teeth, by Mr. Fox. —— These several Lectures are so arranged, that no two of them interfere in the hours of attendance; and the whole, together with the Lectures on Anatomy, and those on the Principles and Practice of Surgery, given at the Theatre of St. Thomas's Hospital, is calculated to form a complete Course of Medical and Chirurgical Instruction.

Mr. Moor, Surgeon Dentist to her Royal Highness the Duchess of York, will commence a Course of Lectures on the Structure and Diseases of the Teeth, on the second Thursday of February, in which will be explained the Complete Practice of the Dentist. Further particulars may be known at his house, No. 6, Palsgrave Place, Temple.

